# Strengthening of Concrete Element with Precast Textile Reinforced Concrete Panel and Grouting Material

**DOI:** 10.3390/ma13173856

**Published:** 2020-09-01

**Authors:** Young-Jun You, Hyeong-Yeol Kim, Gum-Sung Ryu, Kyung-Taek Koh, Gi-Hong Ahn, Se-Hoon Kang

**Affiliations:** Structural Engineering Department, Korea Institute of Civil Engineering and Building Technology (KICT), Goyang 10223, Korea; yjyou@kict.re.kr (Y.-J.Y.); ryu0505@kict.re.kr (G.-S.R.); ktgo@kict.re.kr (K.-T.K.); agh0530@kict.re.kr (G.-H.A.); kshun@kict.re.kr (S.-H.K.)

**Keywords:** carbon textile, flexural strengthening, load-bearing capacity, textile reinforced concrete (TRC), TRC panel

## Abstract

Textile reinforced concrete (TRC) has widely been used for strengthening work for deteriorated reinforced concrete (RC) structures. The structural strengthening often requires accelerated construction with the aid of precast or prefabricated elements. This study presents an innovative method to strengthen an RC slab-type element in flexure using a precast panel made of carbon TRC. A total of five RC slabs were fabricated to examine the flexural strengthening effect. Two of them were strengthened with the precast panel and grouting material and another set of two slabs was additionally strengthened by tensile steel reinforcement. The full-scale slab specimens were tested by a three-point bending test and the test results were compared with the theoretical solutions. The results revealed that the ultimate load of the specimens strengthened with the TRC panel increased by at least 1.5 times compared to that of the unstrengthened specimen. The application of the precast TRC panel and grouting material for the strengthening of a prototype RC structure verified its outstanding constructability.

## 1. Introduction

Textile reinforced concrete (TRC), additionally referred to as fabric-reinforced cementitious matrix, is concrete strengthened with textile made by a multi-axial arrangement of high strength fibers such as carbon fiber [1]. Research on TRC systems over the past two decades has led to significant progress in strengthening in flexure of concrete structures with these systems [2]. Several research groups examined the strengthening effect of TRC on the flexural performance of reinforced concrete (RC), considering mostly the fiber type, number of textile plies, and matrix strength as parameters [3,4,5,6,7]. Recently, the effect of mechanochemical activation of the cementitious suspension and the use of various superplasticizers has been studied for binders [8].

A major advanced feature of the precast TRC elements over the RC elements is light weight due to minimum concrete cover thickness. A 9.9 m wide and 60 mm thick TRC roof structure was fabricated and used as a roof for parking structures [9]. More recently, a 4.5 m long and 40 mm thick precast ceiling element has been constructed in which a 70% reduction of deadload was realized when compared to an RC application [10]. Among the precast TRC applications, a precast TRC panel was introduced for structural strengthening of masonry walls [11].

In general, the flexural strength of the RC member is strengthened by an increase of TRC when using high tensile strength fibers such as carbon fiber or when augmenting the number of textile plies. The cementitious matrix constituting TRC exhibits relatively lower tensile properties compared to those of the carbon fiber applied as a reinforcing material. Accordingly, the strength of the matrix is known to have an insignificant effect on the flexural performance of the TRC-strengthened member.

For the reinforcement to fulfill its role in the structural system, sufficient bond strength should be secured to allow monolithic behavior of the reinforcement and matrix. When carbon fiber is chosen as a textile reinforcement for TRC [12], the smoothness of the carbon fiber surface necessitates additional measures to secure the bond strength [13]. Some research groups conducted studies on the change in the bond performance according to the textile surface treatment and concluded that such surface treatment was effective in improving the bond strength of the textile [7,13,14,15].

Strengthening by the TRC system is usually carried out on site by finishing or shotcreting, but these methods need to be improved to widen their applicability. Since TRC is composed of textile as reinforcement and a matrix, most practitioners achieve TRC by installing the textile on the structure to be strengthened and placing the matrix over it. However, this approach should be modified for sites requiring accelerated construction or presenting a narrow working space. For example, accelerated construction may be realized by using precast TRC panels. Recently, a research group in the Korea Institute of Civil Engineering and Building Technology (KICT) [16] fabricated precast TRC panels and proposed a method of applying stay-in-place formwork during the erection of an open-type wharf structure. More recently, the behavior of RC slab-type elements strengthened with a cast-in-place TRC system was experimentally investigated in KICT [17].

The main objective of this study is to propose an innovative strengthening method with a precast TRC panel and grout material for deteriorated or structurally deficient RC structures. This study fabricated four RC slab-type elements strengthened for flexure using grouting material on precast panels composed of a carbon TRC system and subjected them to a three-point bending test. Two of the specimens were strengthened using a TRC panel and grouting material only. Two other specimens were arranged with additional tensile steel reinforcement during the strengthening assuming a sectional loss caused by steel corrosion. The load-deflection behavior of the strengthened specimens is compared and the failure mode is also compared with respect to the unstrengthened specimen and the specimen strengthened with the cast-in-place TRC system. The constructability of the method using TRC panels and grouting material is examined through partial strengthening of a deteriorated RC box culvert.

## 2. Experimental Program

### 2.1. Materials of TRC System

**Figure 1:**
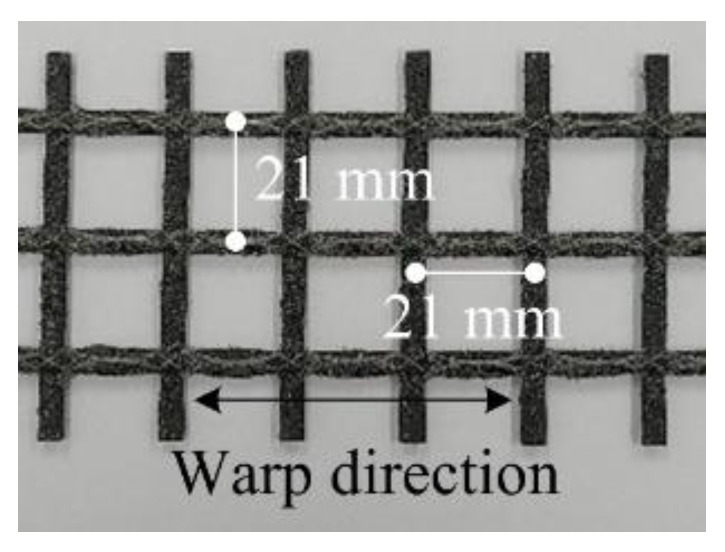
Carbon textile grid.

TRC is composed of a matrix to maintain its shape with the textile reinforcement and to form the structural system. The most common textile reinforcement for the flexural strengthening of concrete structures is the two-dimensional grid shape textile [2,16,18,19]. In this study, the carbon textile grid (Q85/85-CCE-21, Solidian GmbH, Albstadt, Germany) shown in Figure 1 is employed. The mechanical properties of the textile grid are listed in Table 1. The surface of the textile is coated by quartz sand with grain size of 0.3–0.8 mm to improve the bond strength with concrete [20].

Table 2 arranges the mix composition of mortar used in the TRC system. Apart from superplasticizer, this mix composition is identical to that used in a previous study [16]. Polyvinyl alcohol (PVA) fibers (KURALON K-II REC100L, Kuraray, Tokyo, Japan) are admixed at 1% fiber volume fraction in the matrix to prevent the occurrence of cracks induced by the drying shrinkage during the curing process following the fabrication of the TRC panel. The purpose and process of the mix design of mortar for the TRC panel are explained in detail in a previous study [16]. The compressive strength of mortar measured at 28 days on cubic samples is 75.7 MPa.

### 2.2. Fabrication of TRC Panel

**Figure 2:**
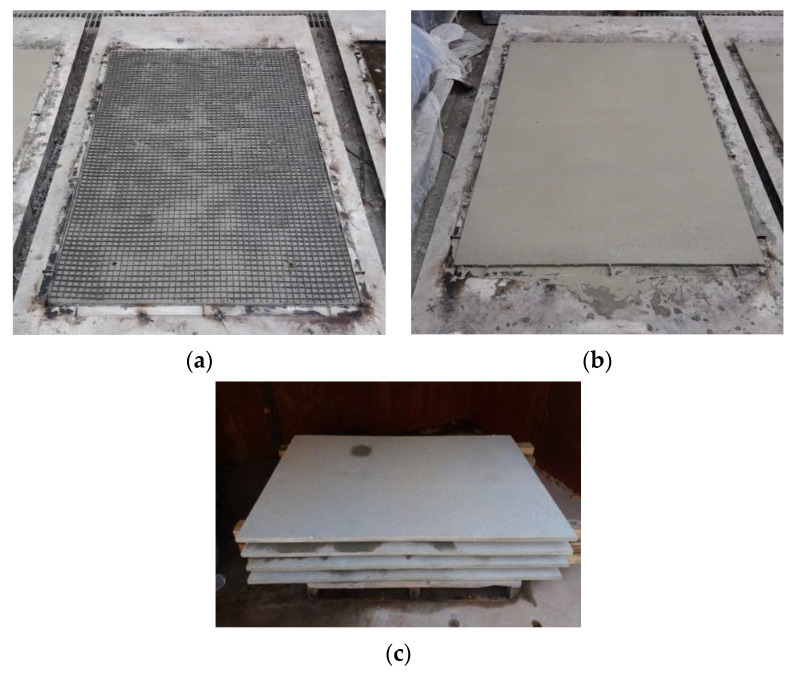
Fabrication process of precast TRC panel: (**a**) 1st mortar layer and textile grid placement; (**b**) 2nd mortar layer placement; (**c**) cured TRC panels.

Figure 2 illustrates the fabrication process of the precast TRC panel. A steel form was first manufactured with dimensions of 20 × 1000 × 1600 mm^3^ (height × width × length) on the steel bedding. Fresh mortar was then placed up to half of the TRC panel thickness and spread evenly prior to putting one ply of textile grid in place, as shown in Figure 2a. Thereafter, the remaining thickness of mortar was placed, finished, and finally naturally cured (Figure 2b). Figure 2c shows the precast TRC panels after completion of curing. Spacers to protect the textile layer from sinking during the fabrication were not used because the fresh mortar was dense. However, if the fresh mortar is not dense, spacers may need to be used to hold the grid during the fabrication.

### 2.3. Fabrication of Full-Scale Slab Specimens

The five RC specimens listed in Table 3 were fabricated for the purpose of strengthening the RC slab with the precast TRC panel. The RC specimen is the control specimen. The SP series indicates the RC slabs strengthened only with the TRC panel. The SSP series stands for the SP specimens in which additional steel reinforcement was arranged assuming section loss due to steel corrosion. Note that the RC slab was designed as an under reinforced slab with a tensile reinforcement ratio of 0.0062. This is because the main objective of this study is to examine the TRC strengthening effect. It should be further noted that the total thickness of the SP series and SSP series specimens is 20 mm greater than that of the RC specimen (control) due to the TRC panel thickness. Therefore, the flexural capacities of the SP series and SSP series specimens should be greater than that of the RC specimen regardless of the strengthening with the TRC panel.

**Figure 3:**
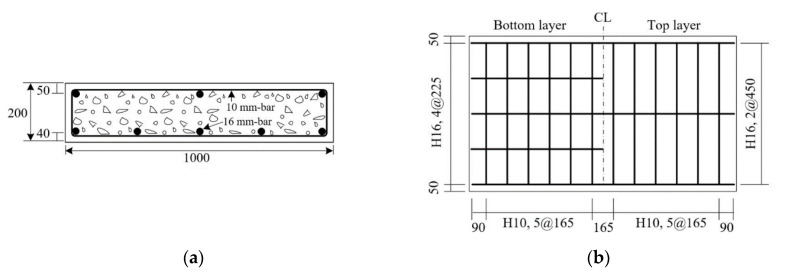
RC slab: (**a**) cross-sectional view; (**b**) reinforcement details (units: mm).

**Figure 4:**
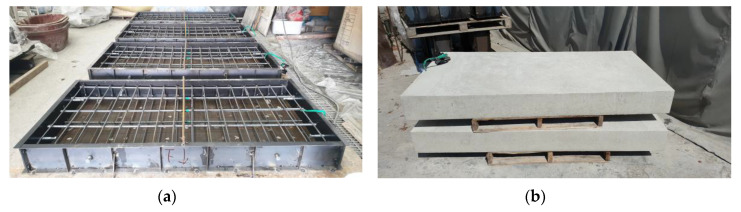
Fabrication of full-size slab specimens: (**a**) steel reinforcement; (**b**) cured specimens.

The dimensions of the full-scale RC slabs are 1000 × 200 × 2000 mm^3^ (width × height × length). As shown in Figure 3, five steel bars and three steel bars with a diameter of 15.9 mm (H16) were arranged respectively at the bottom and top of the slabs, and stirrups with a diameter of 9.53 mm (H10) were arranged for shear reinforcement. The mix composition of ready-mixed concrete used for the fabrication of RC slabs is provided in Table 3. Figure 4a,b depict the completed specimens before and after placing of concrete. The compressive strength measured on a cylinder made of concrete used in the fabrication of the specimens was 33.5 MPa at the age of 30 days.

**Figure 5:**
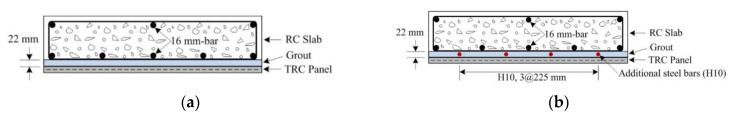
TRC panel strengthening plan: (**a**) SP series; (**b**) SSP series (units: mm).

Four of the completed RC slabs were strengthened with TRC panels. For the SP series specimens (Figure 5a) it was assumed that the bottom face of the RC slab had deteriorated and they were strengthened using only a TRC panel and grouting after having been chipped to the surface of the tensile reinforcement. The SSP series specimens (Figure 5b) simulating the need for additional steel reinforcement following assumed degradation of the bottom side and section loss due to steel corrosion were reinforced by additional steel reinforcement. Additional steel reinforcement with a diameter of 9.53 mm (H10) was placed between the concrete substrate and the TRC panel (Figure 5b).

**Figure 6:**
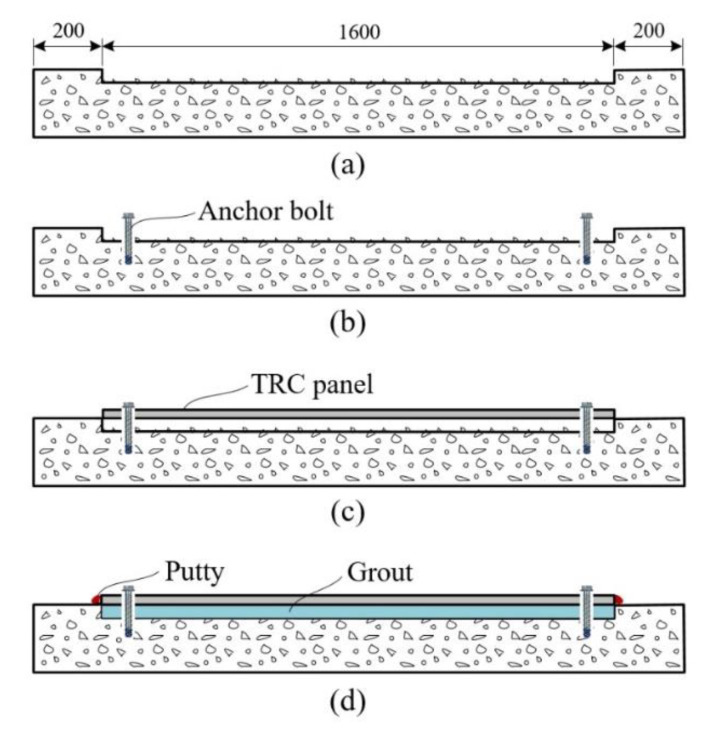
Strengthening process: (**a**) concrete surface chipping; (**b**) anchor bolts installation; (**c**) TRC panel assembling; (**d**) putty placement and grouting.

Figure 6 illustrates the strengthening process using a TRC panel. Strengthening was carried out for a 1600 mm section covering 800 mm on each side of the centerline of the slab. The RC slab was fabricated by disposing its bottom side (tension zone) upward. Figure 6a shows the cured RC slab in which the tensile zone is chipped to a depth of 20 to 25 mm. The anchor bolts are then installed (Figure 6b) and the precast TRC panel is put in place (Figure 6c). In Figure 6d, grout filling is done via putties prepared on both ends of the TRC panel for injecting the grout. The mix composition of the grout is provided in Table 4. The compressive strengths of the mortar used for the fabrication of the TRC panel and the grout used for the TRC panel strengthening are respectively 75.7 MPa at the age of 36 days and 63.7 MPa at the age of 30 days, as measured on cubic samples.

**Figure 7:**
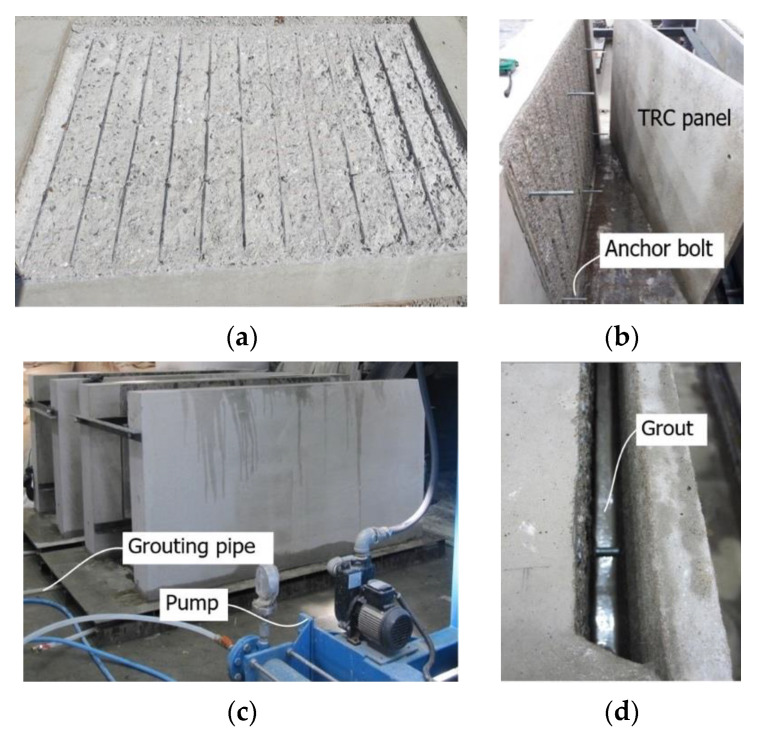
Fabrication of SP and SSP series specimens: (**a**) concrete surface chipping; (**b**) TRC panel assembling; (**c**) grouting pumping; (**d**) grouting.

Figure 7 depicts the fabrication process of the SP series and SSP series specimens. In Figure 7a, chipping was done on the surface of the RC slab until exposure of the tensile reinforcement. Assuming the TRC panel will be constructed on a concrete wall, the slab was set vertically after the installation of the anchor bolts and the TRC panel was assembled with the chipped surface (Figure 7b). Putties were then prepared for the grout filling of the interface between the panel and the slab. Water was sprayed on the surfaces of the concrete slab and TRC panel and the surfaces were maintained in a humid state for 24 h. A grout pumping pipe was connected to the base of the specimens (Figure 7c) and grout filling proceeded from the bottom of the specimens (Figure 7d).

### 2.4. Test-Set up

**Figure 8:**
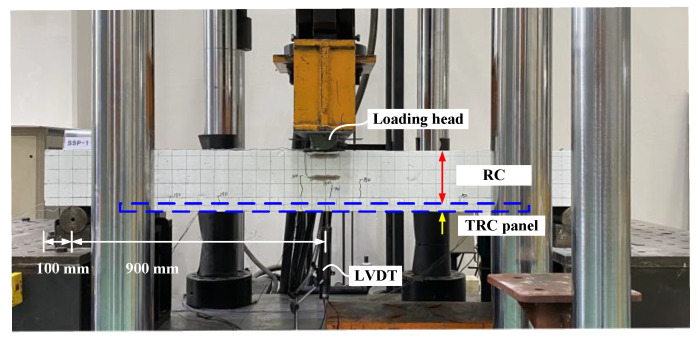
Test set-up for three-point loading.

The flexural performance of the specimen strengthened with the precast TRC panel and grout material was evaluated by a three-point bending test, as shown in Figure 8. Two LVDTs (CASKOREA, Seoul, Korea) were installed along the centerline at the bottom of the slab and loading was applied using a UTM with a capacity of 2000 kN (Daekyung, Seoul, Korea) through displacement control at a speed of 1 mm/min.

## 3. Test Results and Discussion

### 3.1. Load-Displacement Behavior

**Figure 9:**
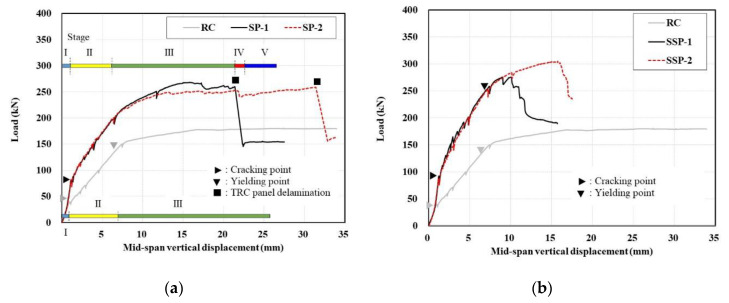
Load-displacement curve of TRC panel reinforced specimens: (**a**) SP series; (**b**) SSP series.

Figure 9 plots the load-displacement curves measured in the bending test of the specimens. The RC specimen (control) exhibits typical behavior of an RC flexural member. The load-displacement curve is quasi-trilinear with a linear part until the initiation of tensile cracks in concrete as the first stage, a load resisting part until the yield of steel reinforcement of unstrengthened cross section in the second stage (see yielding point in Figure 9), and an increase of the displacement only after yield of the steel reinforcement in the third stage. For both the SP and SSP series specimens, the strain gauges were mounted on the top and bottom reinforcements (16 mm bars, Figure 5) only. Hence, the yielding of reinforcement indicates the yielding of the bottom (tensile) reinforcement (16 mm bar). However, it should be noted that the additional steel reinforcement (10 mm bar) might yield first due to a higher depth relative to the neutral axis.

Sets of two specimens were fabricated and tested for each SP and SSP series. Some difference can be observed for the peak load but the behavior and response until the peak load are practically identical. This similarity indicates that the tests were consistent with each other. In view of the load-displacement behavior of the specimen strengthened with the TRC panel, there is some difference in the responses, but the first and second linear behavioral stages between the initiation of concrete cracks and the rebar yield can be distinguished, similarly to those observed in the control RC slab. The difference occurs in the part from the third stage following the second stage after the rebar yield to the peak load. In this part, the specimens of SP and SSP series exhibit a shorter post-yield ductile section than the RC specimen and show a steep reduction of the load-carrying capacity due to sudden failure (fourth stage). In particular, in Figure 9a, delamination between the TRC panel and the concrete occurred in the SP series to lead to sudden failure. In the final fifth stage, the displacement increased continuously without further resisting of the applied loading.

When the RC slab is to be designed for strengthening with the TRC panel, the occurrence of brittle failure after the peak load should be considered carefully. Since the sudden failure happens after the yield of the steel reinforcement of the slab, the structure may be assumed to maintain its load-carrying capacity until the yield of the tensile reinforcement if conservative design is conducted.

In this study, the mortar for the TRC was reinforced with PVA short fibers to mitigate shrinkage-induced crack formation. The PVA short fibers were very effective in preventing shrinkage-induced cracks during the curing of the TRC panel. However, the PVA short fibers did not have a significant influence on the cracking load of the TRC panel and concrete slab in the full-scale flexural failure test.

### 3.2. Load-Carrying Capacity

Table 5 summarizes the bending test results of all sets of specimens. Compared to the RC specimen (control), the load-carrying capacity of the SP series specimens strengthened with the TRC panel only improved by 46% on average and that of the SSP series specimens strengthened with the TRC panel and additional reinforcement improved by a maximum of 69%. Note that the cracking of concrete and the TRC panel occurred simultaneously due to perfect bonding between the concrete substrate and the TRC panel.

Table 6 provides the peak load ratio with respect to the tensile reinforcement ratio for the specimens. The total nominal area of the tensile reinforcement used in the fabrication of the RC slab was 992.8 mm^2^, which gives a tensile reinforcement ratio of 0.0062. The reinforcement ratio of the grid is 0.0004 (=0.085/210), corresponding to a 6.5% tensile reinforcement ratio of the RC slab. Considering that the elastic modulus of the steel reinforcement is 200 GPa, the effective reinforcement ratio is around 7.1% when considering the elastic modulus ratio (1.1 = 220 GPa/200 GPa). In other words, the flexural performance developed up to 146% on average when the tensile reinforcement ratio was increased to 7%. This result can be credited to the high tensile strength of the carbon grid compared to the yield strength of the steel reinforcement.

Four strips of rebar with a diameter of 9.53 mm were additionally arranged in the SSP series. This represents an additional amount (0.0014 = 285.3 mm^2^/1000 × 200 mm^2^) corresponding to a 22.5% tensile reinforcement ratio of the RC slab to be strengthened and means that the reinforcement ratio of the SSP specimens increased by 30% on the whole. In Table 6, the improved resistance of the SSP series reaches 161% on average compared to the control RC slab. If the tensile performance of the grout in the tensile zone is ignored, the best performance was achieved by the installation of the textile grid and steel reinforcement. However, in terms of efficiency, it appears that only strengthening with the textile grid can be recommended. Moreover, the relation between the tensile reinforcement ratio and the load-carrying capacity is not linear.

In view of the behavior after the initiation of cracks and until the yield of tensile reinforcement, the slope (=load/deflection) of the slab strengthened with the TRC panel and that of the slab strengthened with both the TRC panel and additional reinforcement arrangement increased on average by respectively 32% and 58% compared to the unstrengthened RC slab. This indicates that greater load-carrying capacity can be developed by the slab strengthened with the TRC panel under deflection identical to that of the RC slab before strengthening. On the contrary, the RC slab strengthened with the TRC panel experiences smaller deflection than the RC slab before strengthening under application of the same load.

### 3.3. Failure Mode

**Figure 10:**
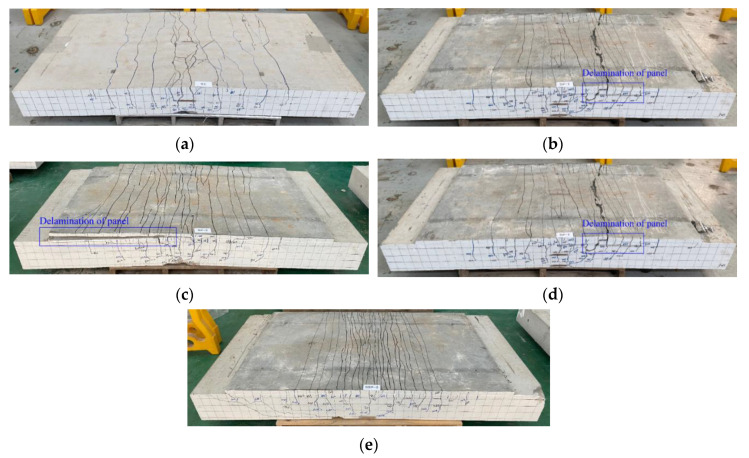
Cracked patterns on the bottom side of specimens after failure (photo): (**a**) RC; (**b**) SP-1; (**c**) SP-2; (**d**) SSP-1; (**e**) SSP-2.

Figure 10 presents the crack patterns of the specimens after completion of the test. The tested specimens were placed in an inverted position to inspect the crack patterns and failure mechanism on the bottom side of the specimens. The RC specimen shows a typical flexural failure mode (Figure 10a). The SP series strengthened with the TRC panel through grout filling exhibited flexural failure and bond failure by delamination of the panel (Figure 10b,c). The SSP series with additional steel reinforcement experienced composite failure mode with the occurrence of failure by the propagation of inclined tensile cracks from the ends of the panel toward the loading point after the initiation of flexural cracking (Figure 10d,e).

The TRC system and the slab must have sufficient bond strength until failure of the slab to develop a sufficient strengthening effect. Accordingly, careful attention should be paid to the bond performance when composing two members. The specimen strengthened with the TRC panel failed through delamination of the panel, but this delamination occurred after the peak load (Figure 9a) and was caused by the excessive curvature experienced by the specimen at failure. Consequently, the TRC panel appears to have achieved sufficient strengthening performance until delamination. It should be further noted that the delamination of the TRC panel from the concrete slab did not occur for the SSP series specimens because the curvature of the SSP series specimens was much smaller than those of the SP series specimens due to the higher effective reinforcement ratio.

In general, the cracking of the RC flexural member changes from cracking induced by pure bending to cracking caused by the combined action of flexure and shear, as the tensile reinforcement ratio is increased [21]. As explained above, the effective tensile reinforcement ratio of the SP series and SSP series was increased respectively by 7% and 30% compared to the RC slab. Therefore, the failure induced by the inclined tensile cracks in the SSP series can be attributed to the significantly larger reinforcement ratio provided by both TRC and additional reinforcement. When the RC flexural member features a high reinforcement ratio, failure is likely to occur suddenly due to the inclined tensile cracks. Accordingly, the effective reinforcement ratio and the failure mode must be checked when design is conducted for strengthening with TRC.

### 3.4. Effect of Strengthening Methods

The TRC system is generally constructed by finishing or shotcreting. Recently, KICT [17] carried out a study on the flexural performance of RC slabs strengthened with a cast-in-place TRC system by mortar shotcreting using an RC slab with the same dimensions as that of the present study and a TRC system with the same thickness as that of the TRC panel of this study. The same carbon fibertex (3200 tex) was used for the textile grid but the grid spacing was 25 mm, which represents a cross-sectional area that is approximately 17% smaller compared to the textile grid used in this study. Polystyrene was used as resin and the tensile strength and elastic modulus were respectively 1700 MPa and 200 GPa. The mix design of mortar used in the TRC system and the strengthening thickness of 20 mm are identical.

**Figure 11:**
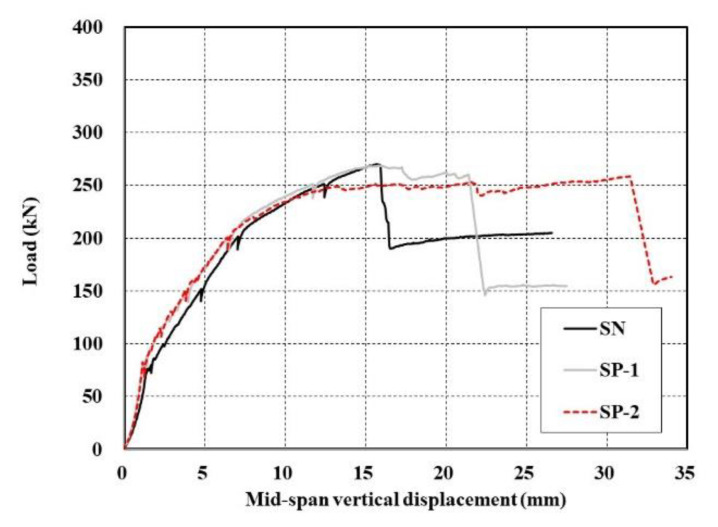
Load-displacement curve for SN and SP series specimens.

**Figure 12:**
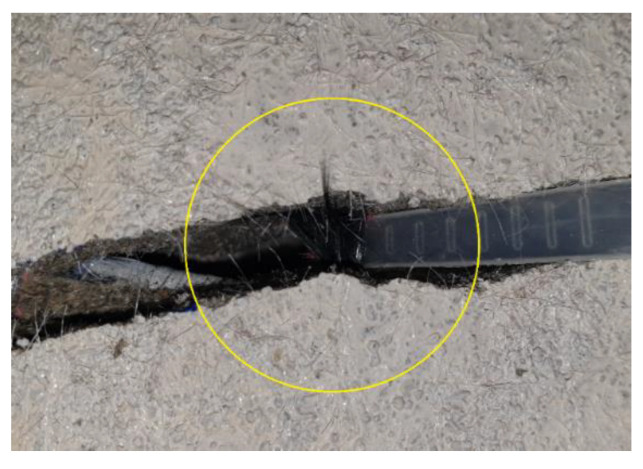
Rupture of textile (SN specimen).

Figure 11 compares the load–displacement curves of the specimen strengthened with the TRC panel and the SN specimen strengthened by a cast-in-place TRC system of KICT [17]. The SP series and the SN specimen exhibited quasi-similar load resisting performance where the load-carrying capacity develops until the peak load and shows a steep loss after failure to reach a level comparable to that of the yield load of the matrix. It can be clearly seen from Figure 11 that the failure of the SN specimen occurred with a low value of displacement relative to that of the SP series specimens because the tensile strength of the textile used for the SN specimen was approximately 51% of that used for the SP series specimens. However, in view of the post-failure strength, the strengthening with the cast-in-place TRC system exhibits a slightly higher value of 200 kN than that (150 kN) developed by strengthening with TRC panels. Considering that the yield load of the RC slab to be strengthened was around 140 kN (Table 5), the specimen strengthened with a TRC panel faithfully fulfilled its strengthening performance after the final failure. The specimen with the cast-in-place TRC system continued to resist the external force to some extent by transferring it to the remaining fibers even when the specimen partially lost its strengthening performance due to the rupture of a portion of the fibers of the carbon grid (Figure 12). Despite that post-failure is meaningless in terms of the resistance of the structure, this transfer of the external force can be credited to the relatively uniform bonding between the TRC and the slab in the specimen with the cast-in-place TRC system.

If the failure of the RC slab strengthened with the TRC panel is not flexural tensile failure of concrete nor concrete crushing on the compression zone, this failure can be attributed to the following four causes. The first cause is the rupture of the textile when the TRC system and the slab are perfectly composed. The second cause is the bond failure between the TRC panel and the slab. The third cause is the shear (inclined tension) of the slab. The final cause is the failure caused by a slip between the carbon grid and the matrix. The first and second causes generally generate brittle failure due to the adopted materials and the combination of two different members. This situation can be verified in Figure 11 through the sudden loss of load-carrying capacity after the peak load in the load-displacement curve of the SN specimen in which some fibers of the carbon grid experienced rupture.

**Figure 13:**
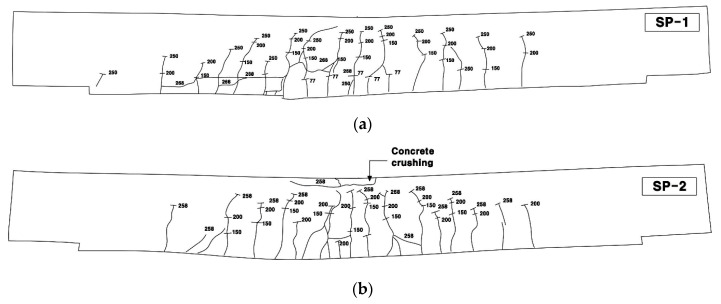

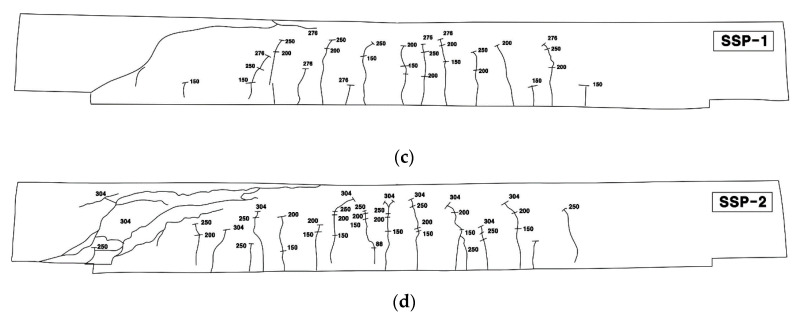
Cracked patterns on the bottom side of specimens after failure (sketch): (**a**) SP-1; (**b**) SP-2; (**c**) SSP-1; (**d**) SSP-2.

Figure 13 shows the crack patterns viewed from the side of the specimens. The failure induced by the inclined tensile cracks tended to be brittle in some cases. For the SP series in Figure 13a,b, inclined tensile cracks were not visibly observed until failure. In addition, for the SSP series in Figure 13c,d, inclined tensile cracks were observed. For SSP-1 specimens, these cracks were not visible until 250 kN but developed immediately when reaching the peak load of 276 kN. In the case of the SSP-2 specimens, inclined tensile cracks were observed at around 250 kN and developed suddenly when the peak load of 305 kN was reached. The occurrence of inclined tensile cracks in the SSP series was caused by the change of the failure mode from flexure to flexure-shear due to the additional arrangement of reinforcement. Even though the slope of the load-displacement curve is relatively smaller than that of the SN specimen (Figure 9), the failure of the RC slab due to the inclined tensile cracks approximately shows the pattern of brittle failure.

The load-displacement curve of the RC slab strengthened only with the TRC panel shows a slightly different shape. In Figure 9 and Figure 11, the displacement measured from the yield load to the peak load appears to be longer than those of the SN and SSP series. This shape appears when the load-carrying capacity continuously decreases as the applied load increases. If inclined tensile cracks do not continuously develop according to the load increase, this situation can be attributed to the occurrence of slip between the matrix and carbon fiber of TRC or to the successive rupture of the carbon fibers forming the textile. In other words, the resistance degrades due to the slip or rupture of carbon fiber and this degradation process repeats whenever the load is increased. This explains the continuous increase of the displacement without any increase in the load-carrying capacity. Since carbon fiber is a brittle material with high tensile performance, the load–displacement curve is likely to exhibit a stepwise shape if the strands undergo rupture successively. Accordingly, the experimental results of this study can be credited to the occurrence of slip between the matrix and the carbon grid constituting TRC. Note that such results are not caused by the difference in the strengthening method; rather, they are attributable to the material properties of the mortar and textile grid.

### 3.5. Analytical Solutions

The theoretical analysis is based upon the following general assumptions adopted in the analysis of RC structural sections [22] together with additional assumptions considering the TRC system: (1) the strain distribution in the section is linear. In other words, a plane section before flexure remains plane after flexure and is perpendicular to the neutral axis; (2) concrete and steel reinforcement as well as the structure being strengthened and the TRC system are perfectly composed and behave monolithically; (3) the resistance of the TRC matrix to cracking is ignored; (4) steel reinforcement behaves bi-linearly and textile behaves linearly; and (5) the effective tensile strain level in the carbon grid is set to 0.012, as recommended by ACI [23]. The theoretical analysis presented in this paper considered flexural failure only.

The ultimate compressive strain of concrete in the compression zone is 0.003 and the following stress-strain behavior is considered for concrete [24]:

(1) $$f_{c}=f_{c}^{\prime }\;\left\{\frac{2\varepsilon_{c}}{\varepsilon_{c0}}-\left(\frac{\varepsilon_{c}}{\varepsilon_{c0}}\right){}^{2}\right\}$$

where $f_{c}$, $\varepsilon_{c}$ = compressive stress and strain levels in concrete; $f_{c}^{\prime }$, $\varepsilon_{c0}$ = maximum stress in flexure of concrete and strain corresponding to the maximum stress of concrete ($1.7\;f_{c}^{\prime }/E_{c}$); and $\varepsilon_{cu}$, $E_{c}$ = ultimate compressive strain and elastic modulus of concrete.

The internal force of concrete when establishing the equilibrium equation for the internal forces in the loaded section can be obtained by Equations (2) and (3).

(2) $$T_{s}+T_{f}=C_{c}$$

(3a) $$T_{s}=A_{s}\;f_{s}$$

(3b) $$T_{f}=NA_{f}bE_{f}\varepsilon_{f}$$

(3c) $$C_{c}=\alpha_{1}\beta_{1}f_{c}^{\prime }cb$$

where $T_{s}$ = tensile force provided by steel; $T_{f}$ = tensile force provided by TRC; $C_{c}$ = compressive force provided by concrete; c = distance from extreme compression fiber to the centroid of steel reinforcement; and b = width of the cross section. $A_{s}$ = cross-sectional area of longitudinal steel reinforcement; $A_{f}$ = area of fabric reinforcement by unit width; $f_{s}$ = steel tensile strength; *N* = number of layers of mesh reinforcement; $E_{f}$ and $\varepsilon_{f}$ = modulus of elasticity and tensile strain of TRC, respectively. The parameters $\alpha_{1}$ and $\beta_{1}$ for computing the equivalent block stress can be obtained as follows [24].

(4a) $$\alpha_{1}=\frac{3\varepsilon_{c0}\varepsilon_{c}-\varepsilon_{c}^{2}}{3\beta_{1}\varepsilon_{c0}^{2}}$$

(4b) $$\beta_{1}\left(c\right)=\frac{4\varepsilon_{c0}-\varepsilon_{c}}{6\varepsilon_{c0}-2\varepsilon_{c}}$$

Ultimate moment and load can be computed according to Equations (5)–(7) respectively as

(5) $$M_{u}=M_{s}+M_{f}$$

(6a) $$M_{s}=A_{s}f_{y}\left(d-\frac{\beta_{1}\left(c_{u}\right)c_{u}}{2}\right)$$

(6b) $$M_{f}=NA_{f}bE_{f}\varepsilon_{f}\left(d_{f}-\frac{\beta_{1}\left(c_{u}\right)c_{u}}{2}\right)$$

(7) $$P_{u}=\frac{2M_{u}}{a}$$

where $M_{s}$ and $M_{f}$ = by member, steel reinforcement, and TRC, respectively; *d* and $d_{f}$ = distance from extreme compression fiber to centroid of tension steel and TRC, respectively; $f_{y}$ = steel tensile yield strength; $c_{u}$, $M_{u}$, and $P_{u}$= distance from extreme compression fiber to neutral axis, flexural strength, and load at ultimate state, respectively; *a* = distance between load and support points.

The theoretical peak load of the specimens was computed (refer to [5] for the calculation process), and Table 7 compares the analytical and experimental results. The corresponding deflection was calculated from the curvature of the changing section according to the load increase. In Table 7, it appears that the theoretical values obtained using the constitutive laws of each material predict with very good accuracy the experimental results for the RC slab. The peak load is predicted with good accuracy for SP series but there is some discrepancy in the displacement between the predicted and experimental values. The theoretical calculation for the SN specimens in which the fiber experienced rupture provided satisfactory prediction of the peak load and displacement. Therefore, it appears that the slip of the textile inside the TRC system occurred as explained for the SP-2 specimen. For the SSP series, the theoretically calculated values are significantly larger than the experimental values. This last observation can be attributed to the fact that TRC could not sufficiently develop the required material performance due to the failure of the RC slab.

## 4. Field Application

**Figure 14:**
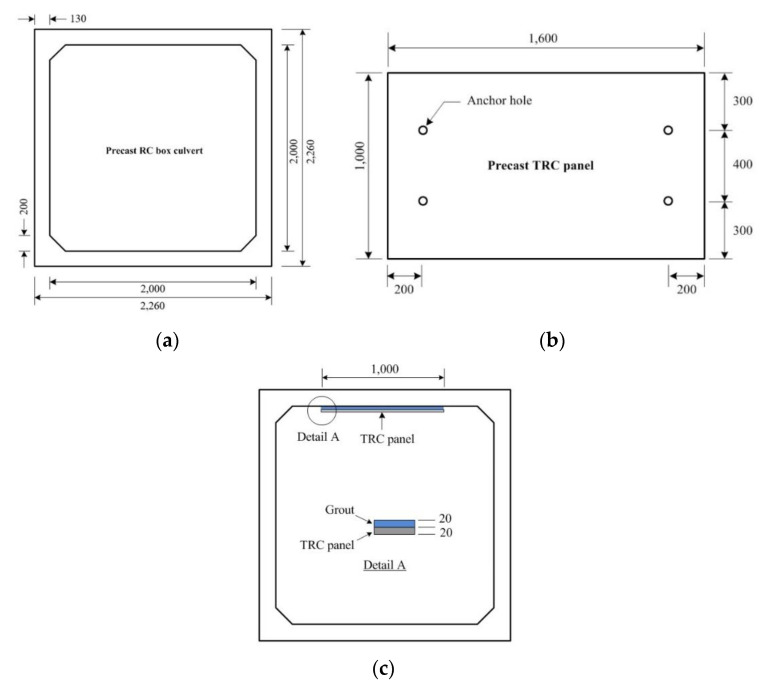
Strengthening plan for a deteriorated RC box culvert: (**a**) dimensions of RC box; (**b**) dimensions of precast TRC panel; (**c**) strengthening plan.

**Figure 15:**
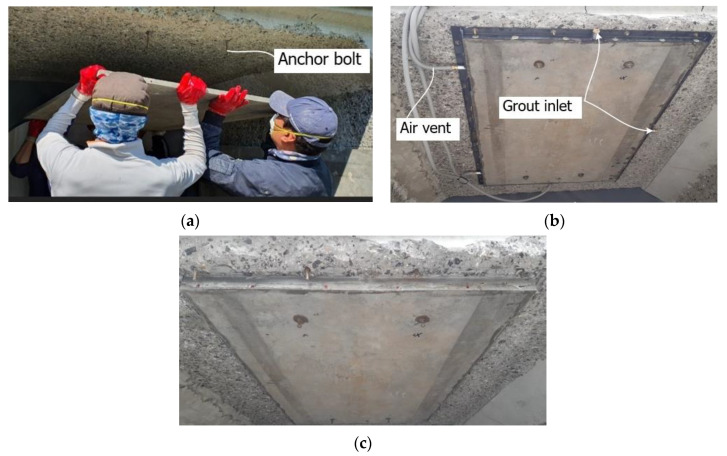
Field application of TRC panel strengthening: (**a**) installation of TRC panel; (**b**) grout pumping; (**c**) finished section.

Trial construction was conducted on a deteriorated RC structure to evaluate the constructability of the flexural strengthening of concrete structures using the precast TRC panel. Figure 14 illustrates the dimensions as well as the strengthening plan for an RC box culvert. As shown in Figure 15a, the deteriorated section at the bottom of the horizontal member of the structure was removed by chipping. Four 100 mm-long chemical anchor bolts (diameter = 6 mm) were installed to fix the TRC panel. A steel form was implemented to encircle the TRC panel and the form was equipped with numerous air vents and inlets to inject the grout (Figure 15b). The grout was then injected under low pressure through the air vents until outflowing. Figure 15c shows the final strengthened section after curing of the grout and removal of the form.

This trial applied the strengthening with a TRC panel on a very small section but verified that accelerated construction could be achieved with outstanding construction quality. In particular, the TRC panel system offers the advantage of being applicable in sites with a very narrow working space or with difficult accessibility.

## 5. Conclusions

This study examined experimentally the load-carrying capacity of RC slabs strengthened with a precast carbon TRC panel. The difference in the performance with a strengthening method using a TRC system was analyzed through a field application and the use of a precast TRC panel. The following conclusions can be drawn.

- When the RC slab is strengthened by the TRC system, the strengthened RC slab experienced brittle failure caused by inclined tensile cracking, rupture of textile, or bond failure of the panel after having developed its yield strength. Accordingly, a conservative design may consider that the load-carrying capacity of the structure is maintained until the yield of the tensile reinforcement.
- When strengthening the RC slab applying TRC, the load-carrying capacity was increased on average by 46% in the case using only the panel and by 61% in the case using the panel and additional reinforcement. The stiffness was also improved. The TRC panel could develop sufficient bond performance to secure monolithic behavior with the RC slab until the peak load. Consequently, the strengthening performance of the TRC system was verified to be effective in terms of the resistance to external force.
- With regard to the improvement of the load-carrying capacity according to the increase of the reinforcement ratio, only strengthening by applying TRC appeared to be preferable to that using both TRC and additional steel reinforcement. There was no linear relationship between the tensile reinforcement ratio and the load-carrying capacity.
- Higher load-carrying capacity and stiffness can be expected when strengthening the RC slab by applying TRC. However, the reinforcement ratio should be checked in the design because the increase of the tensile reinforcement ratio modified the failure mode from flexural failure to composite failure. In particular, careful attention should be paid in the case of a higher reinforcement ratio when failure due to inclined tensile cracks is induced because of the likeliness of brittle failure.
- No attempt has been made to evaluate the long-term performance of the TRC system in this study. However, degradation of the TRC system in certain environments such as under fatigue loading and high temperature could occur. Therefore, evaluating the long-term performance of the precast TRC panel strengthening system under realistic environmental conditions should be another major task of future study.

## Figures and Tables

**Table 1:** Material properties of textile grid in warp direction (suggested by the manufacturer).

Fiber	Resin	Cross-Sectional Area of Yarn (mm^2^)	Tensile Strength (MPa)	Elastic Modulus (GPa)
3200 tex ^1^	Epoxy	1.81	3300	220

^1^ tex = Grams per kilometer of yarn.

**Table 2:** Mixture composition of mortar for textile reinforced concrete (TRC) (unit: kg/m^3^).

Cement	GGBS ^1^	Sand	Water	Superplasticizer
466	466	1024	278	7

^1^ GGBS = Granulated Blast-furnace Slag.

**Table 3:** Characteristic of full-scale slab specimens.

Specimen ID	Total Thickness (mm)	Cross-Sectional Area of Textile (mm^2^/m)	No. of Additional Steel Bars	Remarks
RC	200	-	-	Control
SP-1	220	85.0	-	TRC panel + grout
SP-2	220	85.0	-	
SSP-1	220	85.0	4	TRC panel + grout + steel bars
SSP-2	220	85.0	4	

**Table 4:** Mix composition of ready-mixed concrete (unit: kg/m^3^).

Cement	Water	Fly Ash	GGBS	Sand	Coarse Aggregate	Superplasticizer
263	167	56	56	828	934	2.63

**Table 5:** Test results of failure test for TRC panel strengthened slab specimens.

Specimen ID	Concrete Cracking		Steel Yielding		Failure		Load Gain
	Load (kN)	Displacement (mm)	Load (kN)	Displacement (mm)	Load (kN)	Displacement (mm)	
RC	39.2	0.9	137.6	6.5	180.5	27.1	100%
SP-1	80.8	1.1	201.1	6.4	268.6	15.6	149%
SP-2	82.3	1.1	199.0	6.1	258.7	31.1	143%
SSP-1	92.6	1.2	252.8	7.1	276.1	9.0	153%
SSP-2	85.5	1.2	249.6	7.0	305.0	15.8	169%

**Table 6:** Average load gain relative to control specimen.

Specimen ID	Effective Reinforcement Ratio (%)				Average Load Gain (B)	B/A
	Steel	Textile	Total	Ratio (A *)		
RC	0.0062	-	0.0062	1.00	1.00	1.00
SP series	0.0062	0.0004	0.0066	1.06	1.46	1.38
SSP series	0.0076	0.0004	0.008	1.29	1.61	1.25

* ratio to steel reinforcement of RC specimen.

**Table 7:** Comparison of test data with analytical solutions.

Specimen ID	Experiment		Analysis		Analysis/Experiment	
	Displacement (mm)	Peak Load (kN)	Displacement (mm)	Peak Load (kN)	Displacement	Load
RC	27.1	180.5	27.6	180.6	102%	100%
SN	15.7	270.1	17.9	273.2	114%	101%
SP-1	15.6	268.6	17.9	273.2	115%	102%
SP-2	31.1	258.7	17.9	273.2	58%	106%
SSP-1	9.0	276.1	18.3	378.3	202%	137%
SSP-2	15.8	305.0	18.3	378.3	116%	124%
